# Implementing risk-stratified screening for common cancers: a review of potential ethical, legal and social issues

**DOI:** 10.1093/pubmed/fdt078

**Published:** 2013-08-28

**Authors:** A.E. Hall, S. Chowdhury, N. Hallowell, N. Pashayan, T. Dent, P. Pharoah, H. Burton

**Affiliations:** 1 PHG Foundation (Foundation for Genomics and Population Health), 2 Worts Causeway, Cambridge CB1 8RN, UK; 2 UCL Department of Applied Health Research, University College London, 1-19 Torrington Place, London WC1E 6BT, UK; 3 Department of Public Health and Primary Care, Institute of Public Health, University of Cambridge, University Forvie Site, Robinson Way, Cambridge CB2 OSR, UK; 4 Department of Oncology, University of Cambridge, Cambridge CB2 2QQ, UK

**Keywords:** cancer, consent, distributive justice, personalized screening, risk-stratified screening

## Abstract

**Background:**

The identification of common genetic variants associated with common cancers including breast, prostate and ovarian cancers would allow population stratification by genotype to effectively target screening and treatment. As scientific, clinical and economic evidence mounts there will be increasing pressure for risk-stratified screening programmes to be implemented.

**Methods:**

This paper reviews some of the main ethical, legal and social issues (ELSI) raised by the introduction of genotyping into risk-stratified screening programmes, in terms of Beauchamp and Childress's four principles of biomedical ethics—respect for autonomy, non-maleficence, beneficence and justice. Two alternative approaches to data collection, storage, communication and consent are used to exemplify the ELSI issues that are likely to be raised.

**Results:**

Ultimately, the provision of risk-stratified screening using genotyping raises fundamental questions about respective roles of individuals, healthcare providers and the state in organizing or mandating such programmes, and the principles, which underpin their provision, particularly the requirement for distributive justice.

**Conclusions:**

The scope and breadth of these issues suggest that ELSI relating to risk-stratified screening will become increasingly important for policy-makers, healthcare professionals and a wide diversity of stakeholders.

## Background

Increasing genetic knowledge has identified a large number of genetic variants associated with breast, prostate and ovarian cancers, which occur commonly across whole populations.^1^ Common genetic variants typically confer only marginally increased risks (unlike rare genetic variants such as BRCA 1 or 2, where an adverse genetic test is highly predictive of disease development). However, modelling these more common gene-disease associations provides evidence that genotyping confers additional utility when used with other risk factors,^2,3^ such as age, environmental and lifestyle factors, to identify and target screening of individuals at higher risk of developing disease.^4–6^ The COGS project, an integrated European Union FP7 funded research programme combining elucidation of common genetic variants, modelling and ethical, legal, social and organizational issues analysis (ELSI), held a series of international, multidisciplinary workshops to identify and scope relevant issues arising from risk-stratified screening using genotyping. This paper analyses these and other ELSI issues in terms of four bioethical principles: respect for autonomy, beneficence, non-maleficence and justice, comprising Beauchamp and Childress's bioethical framework, which is widely used in health policy (Appendix 1).^7^

The benefit of risk-stratified screening incorporating genotyping (hereafter risk-stratified screening) is that individuals could be offered tailored interventions (where screening frequency, overall duration and modality vary). This means that risk-stratified screening could result in fewer screening episodes whilst largely maintaining early cancer detection rates and helping to mitigate the harms associated with screening programmes such as overdiagnosis and unnecessary treatment.^6,8^ Applying risk assessment using low-penetrance susceptibility variants to individuals within an entire population would allow the systematic designation of different strata or risk groups, each with different packages of care.^9^ For example, those at lowest risk might forgo screening altogether, thereby avoiding the associated risks of the screening procedures, whilst those at highest risk might commence screening earlier and stop later—and be offered interventions such as X-ray mammography at shorter inter-screening intervals during the screening period.

Despite the theoretical advantages of adopting a risk-stratified approach, such programmes are far from implementation: scientific, technical and operational details still need to be resolved.^10^ Consequently, empirical evidence demonstrating how a risk-stratified approach might reduce the numbers screened and the associated harms (such as overdiagnosis and false-positive findings) is not yet available. However, with evidence mounting of the potential utility of risk-stratified screening for common cancers (such as breast and prostate), policy-makers, health professionals and other stakeholders need to consider the ethical, legal and social issues (ELSI) that might arise.

### What form might risk-stratified screening for common cancers take?

**Fig. 1:**
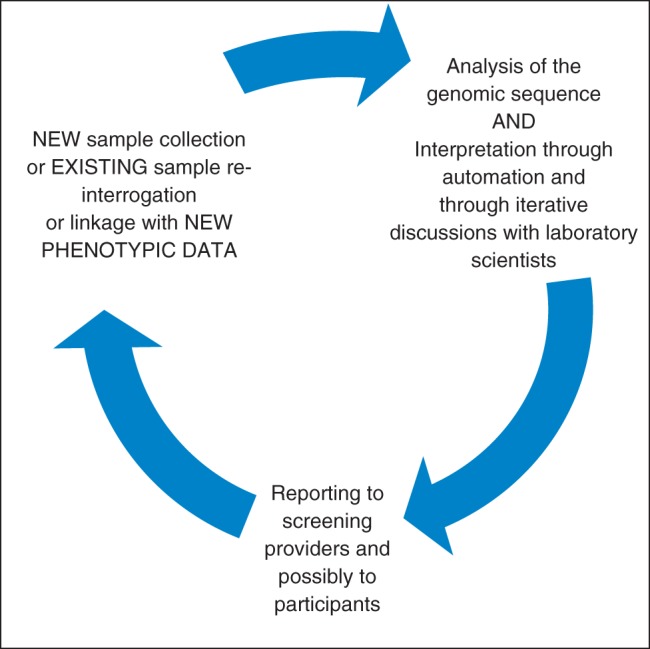
The iterative process of sample and data collection and analysis.

The ELSI issues arising are partially contingent upon the form of risk-stratified screening adopted and the manner of its implementation.^2,5^ Common to all forms is an iterative process of DNA sample and data collection, interpretation and reporting as described in Fig. 1.

The type of samples collected and the way they are stored and interpreted (including whether personal identifiers are removed) will influence the ELSIs that arise. We sketch two alternative data collection and storage scenarios in Table 1, suggesting some relevant ELSI. In reality, these issues would be more complex and dependent upon context.

**Table 1:** Illustrations of databases used for genotyping common cancers

*Illustrative characteristics*	*Model A*	*Model B*
	*Targeted*	*Generic*
	*Single use/disposal*	*Multiple use/retention*
Number of conditions	Single	Multiple
Number of SNPs	10s	100s
Type of sample	Buccal/blood	Blood
Storage conditions	Fresh	Frozen
Storage duration	Days	Many years
Nature of data	Sensitive personal data	Sensitive personal data
Extent of anonymization	Data likely to be personal identifiable data stored and accessed for immediate use	Data likely to be stored as linked anonymized data
Decision support tool for sample and data donors	Unlikely	Possible
Nature of the consent	Likely to be broad consent (perhaps implied from context of care)	Likely to be explicit/specific consent
Need to accomodate changes in capacity to consent (such as child maturing to an adult, or loss of capacity through illness or disability)	Unlikely	Likely
Possibility of withdrawal	Unlikely	Opportunities and mechanisms for withdrawal should be formalized
Breadth of clinical question	Narrow	Broad
Disclosure of incidental information	Clinical question is circumscribed/targeted so less probability of incidental information being generated	Consent should be sought for feedback of incidental information, and mechanism/process should be clear
Reinterrogation/future use for proband	—	Yes
Future use for family members	—	Yes if consented
Third-party use for research (including epidemiological research)	Possible use of anonymized samples and data only	Yes if consented
Access by insurers/employers	Unlikely. Insurers/employers may use surrogates (e.g. invitation to screening instead)	Yes if consented
Re-contact (e.g. for additional testing or to update risk assessments)	—	Yes if consented

In Model A, genotyping is performed as an adjunct to a targeted screening programme for a single disease. Samples are collected and promptly analysed for the sole purpose of using risk stratification to allocate to risk groups and are then routinely destroyed retaining only the resulting risk score. In Model B, genotyping is established as an ongoing public health and healthcare resource to be used when necessary during an individual's lifetime, with comprehensive coverage across multiple diseases. Since genotypic and phenotypic data are retained for multiple purposes, more robust and comprehensive systems need to be adopted to safeguard data security, and also to provide an infrastructure for dealing with issues such as the need for re-contact, incidental or unsolicited findings or changes in capacity to consent.

### What resultant ELSI issues arise?

Significant ELSIs are potentially raised by adopting risk stratification through Models A and B. Relevant ethical issues include the duration of storage of samples and/or identifiable personal data, safeguards taken to secure privacy and confidentiality, linkage with other phenotypic data, including lifestyle data, and interrogation and possible re-interrogation over a person's lifetime. Re-interrogation might be needed to address changing circumstances such as the discovery of new genetic variants, and alterations in lifestyle risk factors, and may necessitate re-contacting the participant. This possibility should have been raised explicitly at the time samples were taken. Other significant factors include the time elapsing between each genotyping phase and the role of automation and use of algorithms to refine test sensitivity and specificity, and safeguard quality assurance. We consider the extent to which the principles of respect for autonomy, beneficence, non-maleficence and justice are satisfied.

### Respect for autonomy

The ethical justification for seeking consent is that an individual should understand what is proposed and its consequences.^11^ Involvement in this process signifies respect for individual autonomy, namely ‘the ability to choose for oneself how one is to live’.^12^ Additionally, certain discrete elements must be satisfied to ensure legal validity, namely that participants can understand, retain, use or weigh up the information needed to make a decision or communicate their wishes.^11,13,14^ The consent process for risk-stratified screening will therefore need to reflect: the breadth of testing, conditions screened for, any potential for incidental findings, the possibility of, and justifications for re-contact,^15^ and implications for insurance and employment. It should be flexible enough to accommodate individuals opting in and out, to incorporate additional information about risk determinants (including recently discovered genetic variants) and be capable of accommodating patient-centric automated tools to guide effective consent choices,^16^ including changes in competence to consent with increasing maturity.^17^

Thus, the consent process for Models A and B will differ regarding the information needed before sample collection, the availability of decision-support tools and whether consent covers current and/or future use. The ethical issues that arise when consenting to targeted genotyping and risk assessment for a single common cancer as in Model A are relatively modest, particularly if samples are disposed of after use, and there is no ongoing data linkage. However, if samples are collected prospectively, and stored for future genotyping and interrogation for other purposes, as in Model B, consent should address the ownership of samples, data and results.

The consent process may also need to address any potential for incidental or unsolicited findings, and detail how and when these might be fed back to participants, given the emerging consensus within genomic research and biobanking that incidental findings revealing ‘an established and substantial risk of a serious health condition’ should be offered to participants, if clinically actionable.^18^ Although individually common genetic variants confer a small increase in risk, together groups of variants could constitute a substantial risk. The potential for generating incidental findings will be increased in risk-stratified screening if the whole genome is sequenced (perhaps more likely in Model B), or if rare high-risk variants are analysed in parallel with common genetic variants. If data relevant to risk-assessment are retained and improved over an individual's lifetime as in Model B, policies will need to refine how risk prediction information is fed back to prospective screening participants, their health providers or potentially affected family members. Individuals might also be re-contacted to update phenotypic and clinical assessments, and behavioural or environmental risk profiles. Finally, in both Models A and B, individuals would need to understand that secondary use of genetic variant risk information could result in discrimination or stigmatization by third parties, with insurers refusing coverage or charging higher premiums to those at higher risk. Although the UK has a moratorium against disclosing predictive genetic test results to insurers subject to regulatory and financial limits, it expires in 2017, and its scope is limited since the timing and frequency of screening invitations could be used as a proxy for genetic information.^19^ Employers might also use information generated by genotyping to preferentially select or dismiss employees, particularly those at risk from occupational exposures.

### Optimizing beneficence and minimizing non-maleficence in risk-stratified screening

Risk-stratified screening assumes that it is scientifically legitimate to utilize and extrapolate from population-based data to provide individual probabilistic information.^20^ For stratification to result in feasible and clinically useful distinctions between population sub-groups,^5^ any risk-stratification element of a screening programme needs to be flexible enough to incorporate evolving knowledge about genetic variants, and environmental and lifestyle exposures and, if it involves long-term storage (Model B), robust enough to minimize potentially harmful privacy and confidentiality breaches.

One way of minimizing burdens might be to incorporate genotyping within existing population-wide public health screening programmes, such as the newborn screening programme.^21^ Resultant risk assessments could inform decisions about adult-onset conditions including common cancers through population-wide prevention programmes.^22^ Although there is public support for newborn screening for diseases arising in infancy, there are widely divergent views about screening young people for conditions arising later in life. Genotyping children and young people raises many ethical concerns, particularly that genotyping might compromise their future autonomous choices,^23^ concerns addressed by postponing risk assessment and/or targeting young adults at highest risk. Systematic testing could generate many ‘clinically actionable’ findings in younger ‘phenotypically normal’ individuals raising questions about optimal management. In the short term then, the introduction of genotyping of common genetic variants into existing neonatal screening programmes seems unlikely and might overburden providers and overwhelm existing capacity.^24,25^

Another potential harm is that participants may have their confidentiality or privacy breached. Confidentiality may be threatened in both Models A and B if identifiable genetic variant information is disclosed without consent, for example, through linkage with potentially identifying phenotype or lifestyle information. Any re-contact also needs to be carefully managed to avoid confidentiality breaches. However, as common genetic variants occur frequently in the population, disclosure of genotype data alone will rarely enable the source to be identified in the absence of other unique identifiers. Moreover, the low clinical validity and utility of profiles of common genetic variants, restricting their use to risk score generation, suggest that this information is less likely to be used in a discriminatory fashion.^26^

The harms and benefits of gaining predictive genetic knowledge about common genetic variants, and its impact upon behaviour is uncertain: empirical evidence is limited. Systematic reviews of randomized controlled trials have explored how genetic knowledge affects smoking cessation and exercise,^27^ and perceived control.^28^ It is unclear how knowledge about genetic susceptibility to multiple diseases might influence behaviour in the longer term.^26^ Experience of those undertaking direct-to-consumer testing indicates that the psychosocial effects are less profound than predicted,^26^ suggesting that systematic genotyping might be regarded similarly by the wider public.^29^

### The principle of justice: ensuring practice that is fair, equitable and appropriate

The use of genotyping to inform access to risk-stratified screening could exacerbate concerns about distributive justice if some individuals or groups unfairly benefited, for example through their socio-economic status, educational background or ethnicity. These concerns might be mitigated by transparency about the genetic variants forming the evidence base for risk stratification, given that existing modelling relies almost exclusively on studies of Caucasian populations. In addition to undertaking further scientific research on other ethnic populations, screening programmes involving genotyping should recognize the impact of cultural and religious beliefs and practices upon every aspect of sample collection, analysis and storage,^30^ including in some cultures, the importance of the wider family group in decision-making.^25^ Thus, information about the programme and screening interventions should be culturally sensitive, and culturally appropriate support should be provided for different groups of service users to ensure maximum coverage and inclusivity.

One group who may require extra resources and attention are ‘low-risk’ individuals who under a risk-stratified approach may no longer be deemed eligible for screening, or may have a less intensive regimen; some of whom may develop cancer. In order to avoid undermining wider trust in health services, effective communication strategies are needed to ensure that those designated as low risk understand that the rationale in their case for withholding or reducing screening is also to optimize the benefits and reduce screening-related risks. In other words, less screening is about risk reduction not rationing health services.

Recent research has highlighted the importance of genetic solidarity, demonstrated by the collective commitment of individuals to bear costs to help others with a different genotype.^12,31^ Elaborating the genetic differences between individuals through genetic variant testing might undermine a wider sense of genetic solidarity, particularly if cancer screening is available through private providers on a direct-to-consumer basis, irrespective of risk. The availability of multiple providers funded through mixed public and privately funded schemes raises questions about the role of the state in promoting the health of its citizens, particularly the rights and responsibilities owed to vulnerable groups, accessing care through publicly funded schemes. Thus, other aspects of the state's role including regulation, governance and reimbursement might impact upon how fair, equitable and appropriate care is accessed.^32^

### The legal and regulatory framework

As outlined above, it is unclear what model of sample storage may be adopted by risk-stratified screening programmes. Programmes conforming to Model B will have to address the problem of storage and protection of samples/genotypic data. Regardless of whether genotypic information is stored within a generic or dedicated databank that is centralized or localized, publically funded or privatized, safeguards must protect against unauthorized access and data processing, and against privacy breaches. Concerns will be greater if data are readily identifiable, and linked with rich phenotypic data. Whilst targeted use of polygenic variants currently holds limited clinical utility, this might increase, particularly with linkage to emerging personal phenotypic information. Electronic health records might routinize such data transfers whilst raising distinctive challenges in preserving confidentiality, privacy and security.^33^ Another emerging debate which seems likely to intensify as financial pressures increase, is whether providing for the possibility of re-contact might also imply a legal duty to seek out individuals who could benefit from interventions^15,34^ and the right of those individuals to remain in ignorance.

More generally, there is need for harmonization in global governance to manage increasing fragmentation of different elements of sample collection, analysis and interpretation. International safeguards and norms need to be developed that provide a consistent yet flexible approach, taking account of the context for disclosure. Challenges include where disclosure is opposed by the participant, obligations are owed to family members and clarifying requirements to revisit existing data.^35,36^

## Discussion

### Main findings

Genotyping could be used to target public health interventions such as screening and health promotion in the near future. Our assessment of the ELSI issues that might arise suggests that population stratification using genotyping, in combination with a generic model for retaining samples and data for multiple uses over many years, whilst logistically attractive evokes many ethical, legal and social concerns which may preclude such developments. However, as more common variants are identified, and sequencing costs fall, the use of genotyping to guide public health interventions will be increasingly compelling albeit, on an *ad hoc* and localized basis, and will interest a broad range of health professionals and policy-makers.

### What is already known on this topic

This is a rapidly advancing area and, since the COGS project is one of the first to consider ELSI issues arising from risk stratification incorporating genotyping for common cancers, existing knowledge is limited. However, it does seem likely that, as genotyping becomes faster, increasingly accurate and more affordable,^37^ genotyping will be incorporated in risk-assessment processes to address targeted clinical questions and supplement existing screening programmes.

### What this work adds

Our evaluation suggests that ELSIs will significantly impact upon the implementation of risk-stratified screening for common cancers. Future ethical challenges include data security, obtaining a meaningful consent and managing logistical issues around capacity to consent, re-contact, withdrawal and linkage of samples. Given this complexity, if risk stratification involving screening is used to refine the offer of screening then policy-makers may be better advised to adopt a more conservative model (Model A), involving a one-off targeted test plus immediate disposal of the samples and data: in the short term, at least, screening programmes conforming to Model A appear to raise fewer ethical and regulatory challenges.

### Limitations

Given the forward-looking nature of this work, our assessment was based upon an analysis of the literature rather than empirical evidence. Robust policy development will be strengthened by translational research testing the utility of adding SNP analysis to existing predictors such as family history, lifestyle factors and age; empirical evidence about how knowledge of genotype may influence risk perception and behaviour; together with more systematic analysis of the ethical, legal and social concerns that are generated.^38^ Wider political and economic drivers outside the scope of this study may also play a determinative role in the adoption of risk-stratified screening programmes. A critical factor might be whether targeting resources according to risk is perceived as reflecting the interests of the entire screening population.^31^

## Funding

This work was supported by funding from the European Community Seventh Framework Programme under grant agreement 223175 [HEALTH-F2_2009-223175]. The funding source had no role in the writing or submission of the paper for publication for which all authors had responsibility. All authors except N.P. and P.P. are employed by the PHG Foundation (the Foundation for Genomics and Population Health) charitable company registered in England and Wales, charity No. 1118664, company No. 5823194. N.P. is supported by a Cancer Research Clinical Scientist Fellowship. Funding to pay the Open Access publication charges for this article was provided by the European Community Seventh Framework Programme under grant agreement 223175 (HEALTH-F2_2009-223175).

## Appendix 1

**Table:** Principles of biomedical ethics: the four principles^7^

*Bioethical principle*	*Definition*
Respect for autonomy	Acknowledgement of a person's right to hold views, make choices and take actions based on values and beliefs (p. 106)
Non-maleficence	To abstain from causing harm to others (p. 151)
Beneficence	To act for the benefit of others (p. 203)
Justice	Fair, equitable and appropriate treatment in light of what is due and owed to persons (p. 250)
